# Computed Tomography-Guided Transthoracic Needle Biopsy: Predictors for Diagnostic Failure and Tissue Adequacy for Molecular Testing

**DOI:** 10.3389/fmed.2021.650381

**Published:** 2021-05-19

**Authors:** Chia-Ying Lin, Chao-Chun Chang, Chang-Yao Chu, Li-Ting Huang, Ta-Jung Chung, Yi-Sheng Liu, Yi-Ting Yen

**Affiliations:** ^1^Department of Medical Imaging, National Cheng Kung University Hospital, College of Medical College, National Cheng Kung University, Tainan, Taiwan; ^2^Division of Thoracic Surgery, Department of Surgery, National Cheng Kung University Hospital, College of Medical College, National Cheng Kung University, Tainan, Taiwan; ^3^Department of Pathology, National Cheng Kung University Hospital, Tainan, Taiwan; ^4^Division of Trauma and Acute Care Surgery, Department of Surgery, National Cheng Kung University Hospital, College of Medical College, National Cheng Kung University, Tainan, Taiwan

**Keywords:** lung neoplasm, pathology, percutaneous CT-guided transthoracic biopsy, molecular testing, sensitivity and specificity

## Abstract

**Background:** Adequate and representative tissue from lung tumor is important in the era of precision medicine. The aim of this study is to identify detailed procedure-related variables and factors influencing diagnostic success and tissue adequacy for molecular testing in CT-guided TTNB.

**Methods:** Consecutive patients undergoing CT-guided TTNB were retrospectively enrolled between January 2013 and May 2020. Multivariate analysis was performed for predictors for diagnostic accuracy and tissue adequacy for molecular testing. Logistic regression was used to identify risk factors for procedure-related complications.

**Results:** A total of 2,556 patients undergoing CT-guided TTNB were enrolled and overall success rate was 91.5% (2,338/2,556). For lung nodules ≤3 cm, predictors for diagnostic success included coaxial needle use [*OR* = 0.34 (0.16–0.71), *p* = 0.004], CT scan slice thickness of 2.5 mm [*OR* = 0.42 (0.15–0.82), *p* = 0.011] and additional prefire imaging [*OR* = 0.31 (0.14–0.68), *p* = 0.004]. For lung tumor >3 cm, ground glass opacity part more than 50% [*OR* = 7.53 (2.81–20.23), *p* < 0.001] or presence of obstructive pneumonitis [*OR* = 2.31 (1.53–3.48), *p* < 0.001] had higher risk of diagnostic failure. For tissue adequacy, tissue submitted in two cassettes (98.9 vs. 94.9%, *p* = 0.027) was a positive predictor; while male (5.7 vs. 2.5%, *p* = 0.032), younger age (56.61 ± 11.64 vs. 65.82 ± 11.98, *p* < 0.001), and screening for clinical trial (18.5 vs. 0.7%, *p* < 0.001) were negative predictors.

**Conclusions:** Using a coaxial needle, with thin CT slice thickness (2.5 mm), and obtaining additional prefire imaging improved diagnostic success, while obtaining more than two tissue cores and submitting in two cassettes improved tissue adequacy for molecular testing.

## Introduction

CT-guided transthoracic needle biopsy (TTNB) is a widely accepted technique to obtain tissue diagnosis from pulmonary lesions. The procedure has high diagnostic accuracy, ranging 82–98.2% (1, 2), with acceptable complications. In the era of precision medicine, in addition to histopathological diagnosis of tumor subtypes, molecular analysis is also mandatory for personalized and targeted cancer treatment (3, 4). Adequate and representative tissue from the tumor is important for molecular analyses. Furthermore, the expanding clinical trials for cancer patients highlight the importance of tissue samples in determining treatment, evaluating efficacy and characterizing disease progression (3).

Prior studies showed that computed tomography-guided percutaneous fine needle aspiration biopsy of lung lesions yielded high success rate in clinically suspicion of primary lung malignancy, superior lobe location, and larger lesion (≥4 cm) (5). When biopsy for small (≤2 cm) subpleural lesions was to be performed, long transpulmonary needle path (≥1 cm) was associated with higher success rate than short transpulmonary needle path (<1 cm) [93.8 vs. 81.7%, *p* = 0.004; (6)]. When paraffin-embedded tissue is used for molecular testing of lung cancer, core needle biopsy specimens are more likely than fine needle aspiration (FNA) specimens to provide adequate tissue for molecular testing (7).

At present, few large scale studies focus on detailed real world procedure-related predictors of successful sampling and tissue adequacy of CT-guided TTNB. The aim of this study is to identify detailed procedure-related variables and factors influencing diagnostic success and tissue adequacy for molecular testing in CT-guided TTNB.

## Methods

### Study Population

This retrospective study was approved by the institutional review board (A-ER-109-317) and informed consent was waived. Between January 2013 and May 2020, all the patients underwent CT-guided TTNB at National Cheng Kung University Hospital were enrolled as cohort 1 for analysis of success rate. The indicators for TTNB included emerging or unresolved parenchymal tumor mandating definitive diagnosis, focal parenchymal infiltrates in which an infectious organism cannot be isolated, diagnosis of hilar masses following negative bronchoscopy, biopsy or re-biopsy of malignancy for targeted therapy. Coagulation profile was routinely tested before TTNB.

**Figure 1:**
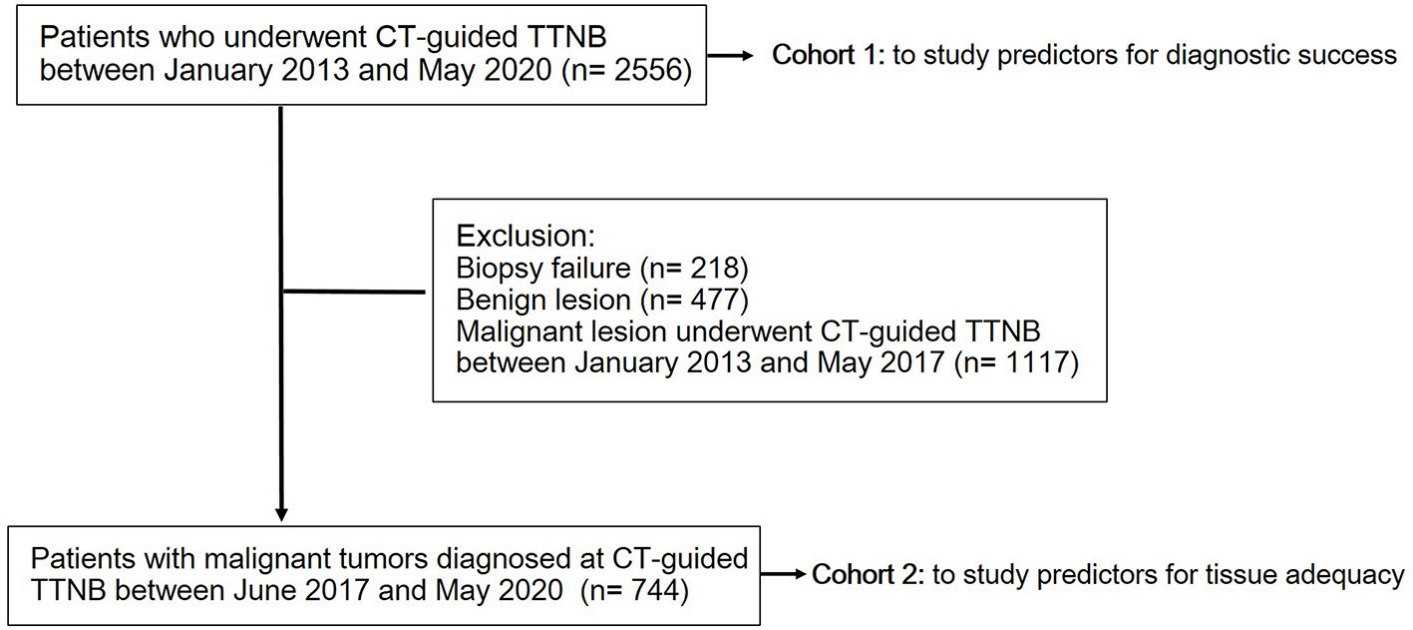
Flow diagram of patient selection.

**Figure 2:**
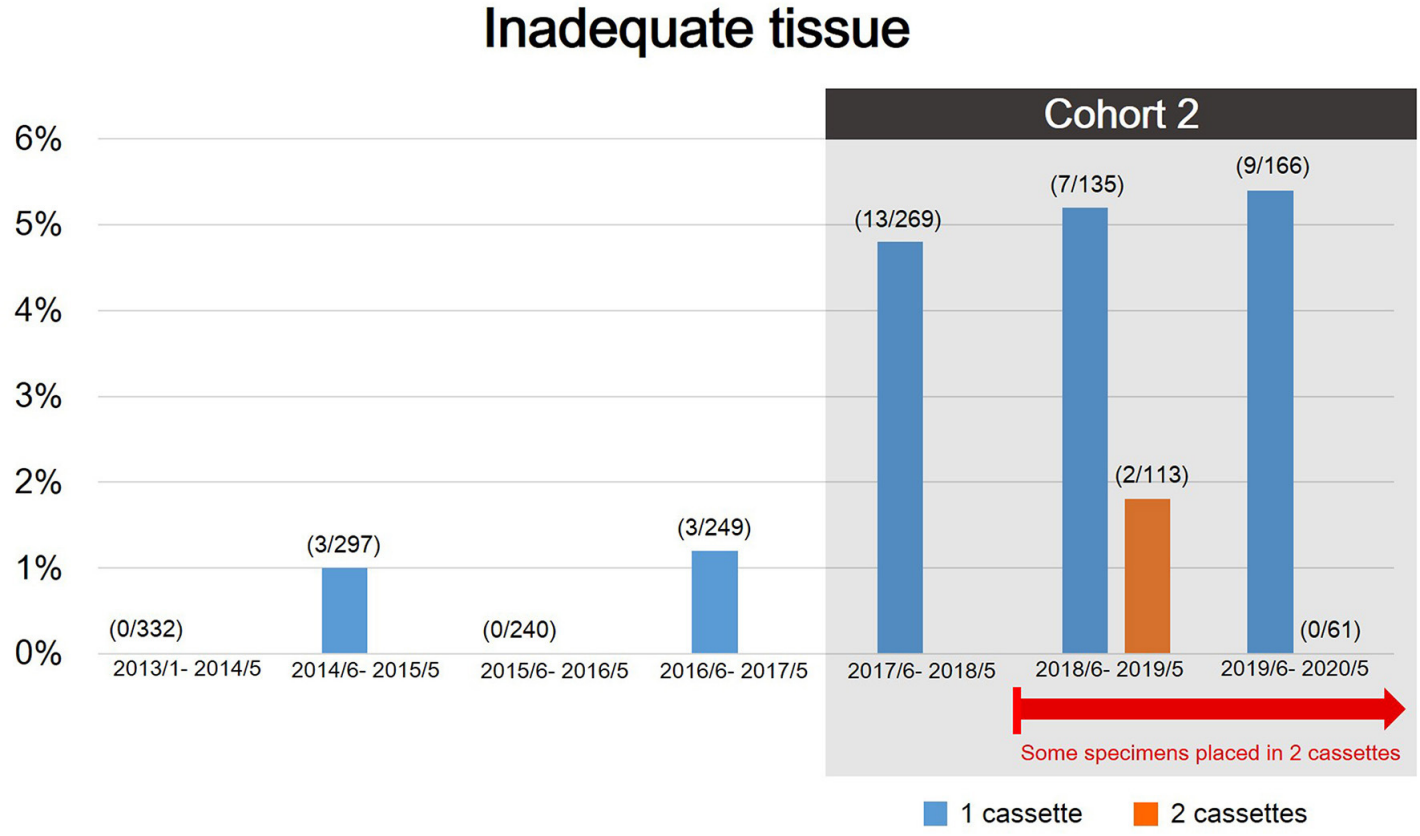
The rate of inadequate tissue during the study period. There was an evident increasing rate of rebiopsy since June 2017 because of expanding molecular analyses and clinical trials. Patients diagnosed with malignancy via TTNB between June 2017 and May 2020 were defined as cohort 2 to further assess predictors for tissue adequacy.

Recently, we found that there was an undesirably high rate of specimens that were suitable for rendering a diagnosis but insufficient for molecular profiling. The rate of re-biopsy increased from <1% to up to 4% since June 2017 due to an increased number of molecular analyses and clinical trials. Therefore, to further delineate the important factors in determining tissue adequacy for molecular testing, we defined patients who were diagnosed malignancy via TTNB between June 2017 and May 2020 as cohort 2, excluding patients with biopsy failure and patients with benign lesion, to assess predictors for tissue adequacy (Figures 1, 2).

### Needle Biopsy Technique

**Figure 3:**
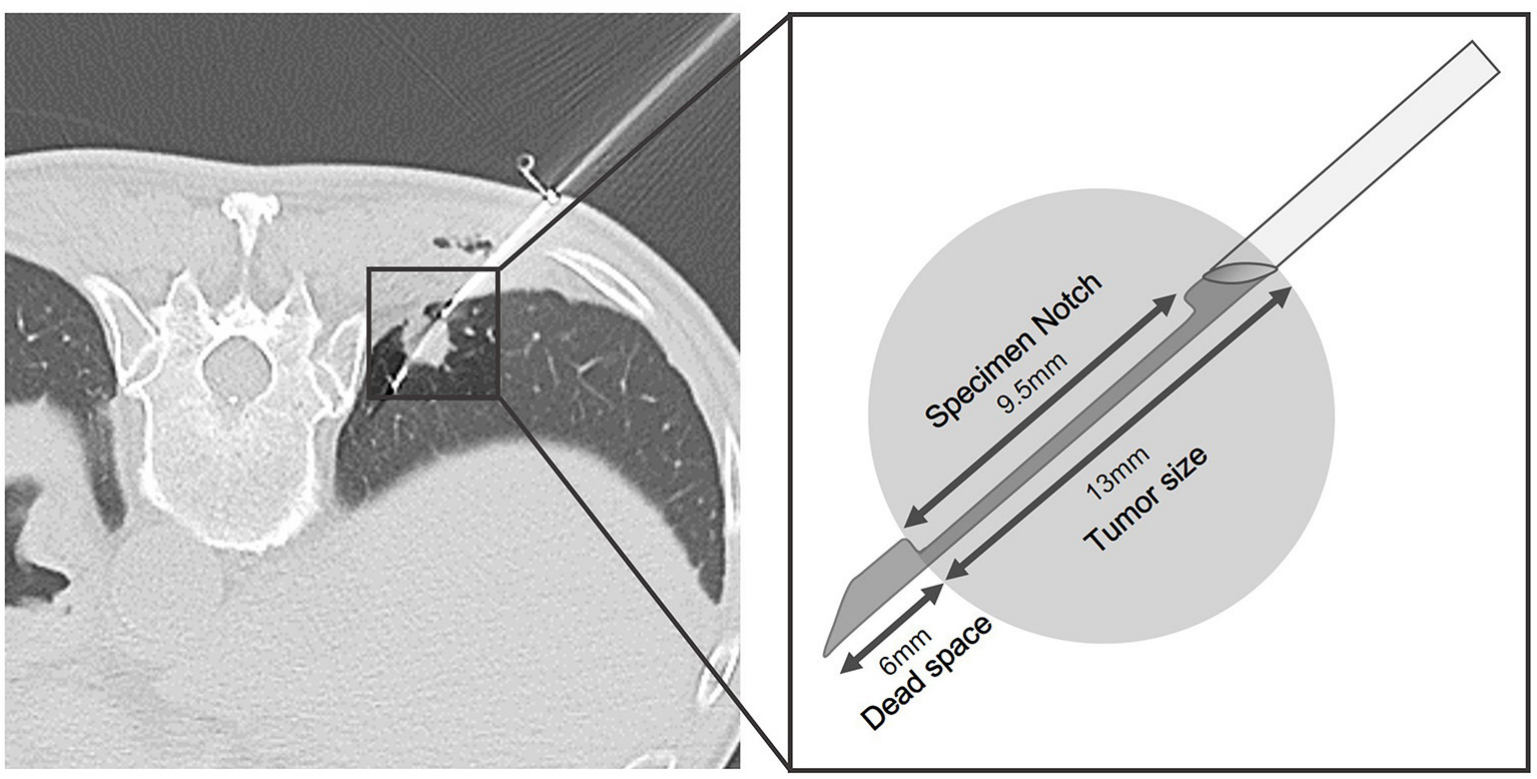
Representative example of additional prefire imaging with schematic illustration in a patient who had colon cancer undergoing CT-guided TTNB for 1.3 cm RLL lung nodule. Before biopsy gun was fired, appropriate specimen notch position (i.e., within the lesion) was confirmed. The pathology result revealed metastasis.

Each biopsy was performed by one of the four board-certificated radiologists (C-YL, T-JC, L-TH, and HLK). The procedures were done without contrast enhancement on a 64-slice CT scanner (Optima 660, General Electric, Milwaukee, WI, USA) with 2.5 or 5 mm slice thickness. Before the procedure, patients were placed in supine, prone or oblique position based on the planned needle trajectory. After patient positioning, local anesthesia, aseptic draping, and biopsy was sequentially performed using single-needle or coaxial technique. The operator subjectively decided the biopsy method. The single-needle technique was performed using an 18- or 20-gauge biopsy needle advancing into the lesion without introducer needle. Once the needle tip reached the lesion and confirmed by CT scan, the specimen was obtained. The coaxial-needle technique was done using a 17- or 19-gauge thin-walled coaxial introducer needle inserted into the lesion, and the 18 or 20-gauge semi-automatic biopsy needle (Tru-core needle; Angiotech, Vancouver, CA, USA) was advanced through the lumen of the introducer needle once the needle was in the lesion. Before the biopsy gun was fired, additional prefire imaging confirming appropriate specimen notch position (i.e., within the lesion; Figure 3) was done at the operator's discretion. Core biopsy specimens were obtained with a 1- or 2-cm needle throw according to tumor size. Biopsy specimens could be obtained by multiple passes of biopsy gun through the coaxial needle system without repositioning the guide needle. The core biopsy specimens were fixed in 10% formalin and then sent to the pathology department. In patients with more than one lung nodules, the choice of which lung nodule to be biopsied was based on the operator's discretion. In general, larger, more solid, peripheral and upper location lesions were favorable choice. After the procedure had been completed, a final set of CT scan was done immediately to detect potential complications, e.g., air emboli or pulmonary hemorrhage. The patient was encouraged to lie still and breath gently during the 4-h recovery period. Chest radiographs were obtained at 2 h after the procedure.

From June 2018, biopsy materials were placed in one or two tissue cassettes based on operator's discretion to minimize loss of diagnostic tissue if IHC (immunohistochemistry) or special stains were used on one of the two blocks, leaving the second block available for ancillary testing.

### Measured Variables

Patient demographics and information were collected on size and location of the lesion, nodule characteristics [including ground glass opacity (GGO) part and cavitary change], presence of emphysema along the biopsy track, presence of obstructive pneumonitis, tumor depth (shortest distance from tumor to pleura), tract distance (distance from skin to lesion along the needle trajectory), use of coaxial needle, size of biopsy needle, slice thickness of CT scan, whether additional prefire imaging was obtained, procedure time, and number of core sample taken. The procedure time was defined as the time interval between the injection of local anesthesia and the final set of CT scan. The percentage of GGO component was classified as more than 50 and ≤50% because the percentage of GGO component reflected invasiveness and growth rate of the tumor (8, 9). The size of tumors was divided into more than 3 and ≤3 cm since the size 3 cm determined T1 or T2 lung cancer (10). The distances of lung parenchymal tract were divided into long (>2 cm) or short (≦2 cm) because lesion-pleural distance of ≧21 mm was identified as a risk factor for complications such as pneumothorax and pulmonary hemorrhage in prior study (6).

Technical success was defined as satisfactory biopsy tissue obtained suitable for histopathologic analysis. After the diagnosis was established, further molecular testing was done using remnant specimen according to NCCN guideline or per clinical study request based on different tumor treatment strategy. Procedure-related complications recorded included air emboli, pulmonary hemorrhage, pneumothorax, and whether drainage tube placement was required. One chest radiologist (C-YL with 7 years of experience) performed all the data collection.

### Diagnostic Criteria

The final diagnosis was confirmed in the following methods: (I) in patient who underwent surgical resection, the final diagnosis was made based on pathologic report of resected specimen; (II) if the TTNB or other biopsy methods revealed malignancies or specific benign abnormalities, the diagnosis was made based on the biopsy results; (III) if non-specific benign abnormality was indicated in a pathology report, the lesion was considered benign if it regressed with medical treatment or remained stable for at least 3 years. Pathologic results were considered inconclusive if malignancies were suspected but not confirmed, including atypical adenomatous hyperplasia (AAH) or atypical cell. Biopsy failure was defined when benign or inconclusive diagnosis turned out to be benign specific diagnosis or missed cancers on subsequent tissue sampling.

### Adequacy for Molecular Testing

Core needle biopsy paraffin-embedded tissue specimens were used once molecular studies were requested. The molecular profiling was done upon request based on the different pathological diagnosis and could varied over time as new testing targets and methods became available. Molecular testing for lung adenocarcinoma, including epidermal growth factor receptor (EGFR) mutation, anaplastic lymphoma receptor tyrosine kinase (ALK) translocation, c-ros oncogene 1 (ROS1) rearrangement or programmed death ligand-1 (PD-L1), was done by the pathology department at our hospital upon request. EGFR mutation analysis was performed with polymerase chain reaction using the amplification-refractory mutation system (ARMS). For ARMS-based EGFR testing, at least 200 tumor cells on formalin-fixed, paraffin-embedded (FFPE) specimens were required for analysis. ALK IHC using D5F3 clones and ROS1 IHC using D4D6 clones were done; in the case of equivocal results, confirmatory FISH testing would be done afterwards. PD-L1 was measured by an approved assay (e.g., PD-L1 22C3 pharmDx method). For unresectable lung squamous cell carcinoma, small cell lung cancer, and adenocarcinoma without driver gene mutation, we performed PD-L1 testing. Additional molecular testing would be performed per clinical trial request. For metastatic tumors from other origins, we performed molecular testing based on treatment guideline or clinical trial requests. The specimens were defined as inadequate if re-biopsy was requested for molecular testing regardless of how many different tests had been done.

### Statistical Analysis

Patient demographics and nodule characteristics were summarized using descriptive statistics (mean ± SD for continuous outcomes, and proportions for categorical outcomes). Chi-square tests, Fisher's exact test, and independent *T*-test were conducted to evaluate the discrete and continuous variables of patient's characteristics. Logistic regression was used to identify risk factors for biopsy failure, inadequate tissue, pneumothorax and pulmonary hemorrhage. A *p* ≤ 0.05 was set to indicate statistical significance. Factors with *p* < 0.05 in the univariate analysis and those considered to be clinically relevant were included in the multivariate analysis. SPSS system (IBM SPSS Statistics, Version 22.0, Armonk, NY) was used for statistical analysis.

## Results

### Patient and Lesion Characteristics

Demographic characteristics were detailed in Table 1. A total of 2,556 consecutive patients (female: male = 1,171:1,385, mean age 63.9 years old, ranging 19–95 years old) who underwent CT-guided TTNB were eventually enrolled for analysis. There were 1,770 (69.2%) primary lung cancers, 275 (10.8%) metastatic tumors, 502 (19.6%) benign lung lesion. Nine (0.4%) patients had no definite diagnosis; 1 was found to have necrotic tissue, 1 was found to have inadequate tissue for diagnosis, 4 were found to have normal lung tissue, and the rest 3 were complicated with air emboli, massive pulmonary hemorrhage, massive pneumothorax during the procedure, respectively. All these 9 patients refused further intervention, and no definite diagnosis was obtained. Of the 1,770 primary lung cancers, 1,524 (86.1%) were adenocarcinoma, 152 (8.6%) squamous cell carcinoma, 70 (4.0%) small cell carcinoma, and 24 (1.4%) others. There were 2,462 (96.3%) tumors with GGO part <50% and 94 (3.7%) tumors more than 50%. There were 1,519 (59.4%) patients found to have more than one nodules.

**Table 1:** Baseline demographic, clinical characteristics, radiologic features, and pathology results of predictors for diagnostic failure of CT-guided TTNB.

**Variables**	**Success (*n* = 2,338)**	**Failure (*n* = 218)**	***p***	Multivariable analysis OR (95%CI)^*^	***p***
Sex			0.263	–	
Female	1,079 (92.1%)	92 (7.9%)			
Male	1,259 (90.9%)	126 (9.1%)			
Age (years)^*^	64.02 ± 13.23	62.27 ± 13.49	0.063		
GGO part			0.061	–	
≦50%	2,257 (91.7%)	205 (8.3%)			
>50%	81 (86.2%)	13 (13.8%)			
Emphysema			0.138	–	
No	2,221 (91.7%)	202 (8.3%)			
Yes	117 (88.0%)	16 (12.0%)			
Obstructive pneumonitis			0.087	–	
No	1,980 (91.9%)	175 (8.1%)			
Yes	358 (89.3%)	43 (10.7%)			
Cavitary change			0.712	–	
No	2,240 (91.4%)	210 (8.6%)			
Yes	98 (92.5%)	8 (7.5%)			
Location			0.145	–	
LLL	382 (90.7%)	39 (9.3%)			
LUL	668 (92.8%)	52 (7.2%)			
RLL	467 (89.5%)	55 (10.5%)			
RML	148 (89.2%)	18 (10.8%)			
RUL	673 (92.6%)	54 (7.4%)			
Tumor depth (cm)	0.65 ± 0.87	0.68 ± 0.92	0.630	–	
**Tumor size**			<0.001		**0.001**
≦3 cm	851 (88.7%)	108 (11.3%)		1 (Reference)	
**>3 cm**	1,487 (93.1%)	110 (6.9%)		**0.602 (0.454**–**0.798)**	
Final pathology			<0.001		
Primary lung cancer					
Adenocarcinoma	1,411 (92.6%)	113 (7.4%)			
Squamous cell carcinoma	132 (86.8%)	20 (13.2%)			
Small cell carcinoma	64 (91.4%)	6 (8.6%)			
Others	18 (75.0%)	6 (25.0%)			
Metastasis	236 (85.8%)	39 (14.2%)			
Benign lung lesion	477 (95.0%)	25 (5.0%)			
No definite diagnosis		9 (4.1%)			
**Coaxial**			0.021		**0.035**
No	1,280 (90.3%)	137 (9.7%)		1 (Reference)	
**Yes**	1,058 (92.9%)	81 (7.1%)		**0.520 (0.283**–**0.955)**	
Biopsy needle			0.698	–	
18-gauge	1,818 (91.4%)	172 (8.6%)			
20-gauge	520 (91.9%)	46 (8.1%)			
Slice thickness			0.009		0.200
≦2.5 mm	290 (95.4%)	14 (4.6%)		0.634 (0.316–1.273)	
>2.5 mm	2,048 (90.9%)	204 (9.1%)		1 (Reference)	
**Additional prefire imaging**			0.001		**0.003**
No	1,329 (89.9%)	150 (10.1%)		1 (Reference)	
**Yes**	1,009 (93.7%)	68 (6.3%)		**0.380 (0.201**–**0.716)**	
Tissue core number			0.012		0.847
≦2	1,911 (90.8%)	193 (9.2%)		1 (Reference)	
≧3	427 (94.5%)	25 (5.5%)		0.945 (0.535–1.671)	
Parenchymal tract distance			0.820	–	
≦2 cm	1,548 (91.4%)	146 (8.6%)			
>2 cm	790 (91.6%)	72 (8.4%)			
Transfissure			0.227	–	
No	2,307 (91.5%)	213 (8.5%)			
Yes	31 (86.1%)	5 (13.9%)			

*Unless otherwise indicated, data in parentheses are percentages*.

* *Data are mean ± standard deviation*.

*The bold values are statistically significant in multivariate analysis, p < 0.05*.

### Predictors for Diagnostic Success

In total, 2,338 patients (91.5%) had successful histologic diagnosis, while 218 patients (8.5%) failed in establishing a histological diagnosis after CT-guided TTNB. There was no difference in age, sex, GGO part of the tumor, presence of emphysema, obstructive pneumonitis, tumor location, depth, cavitary change, biopsy needle gauge, parenchymal tract distance, or transfissure trajectory between the two groups. The predictors for diagnostic success of CT-guided TTNB were shown in Table 1. In univariate analysis, tumor size more than 3 cm (93.1 vs. 88.7%, *p* < 0.001), coaxial needle usage (92.9 vs. 90.3%, *p* = 0.021), CT scan slice thickness of 2.5 mm (95.4 vs. 90.9%, *p* = 0.009), additional prefire imaging (93.7 vs. 89.9%, *p* = 0.001), and tissue core number ≥3 (94.5 vs. 90.8%, *p* = 0.012) significantly increased diagnostic yield. In addition, different pathology results were associated with various diagnostic yield rates. Squamous cell carcinoma, other rare primary lung cancer (e.g., sarcomatoid carcinoma of the lung), and metastatic tumors had higher diagnostic failure rate (*p* < 0.001). In multivariate analysis, tumor size more than 3 cm [Odds ratio (*OR*) = 0.602 (0.454–0.798), *p* = 0.001], coaxial [*OR* = 0.520 (0.283–0.955), *p* = 0.035], and additional prefire imaging [*OR* = 0.380 (0.201–0.716), *p* = 0.003] significantly increased diagnostic yield.

In patients with lung tumor ≤3 cm, the predictors for diagnostic success of CT-guided TTNB were shown in Table 2. In univariate analysis, CT scan slice thickness of 2.5 mm (94.8 vs. 87.9%, *p* = 0.027) significantly increased diagnostic yield. In multivariate analysis, coaxial needle use [*OR* = 0.340 (0.163–0.710), *p* = 0.004], CT scan slice thickness of 2.5 mm [*OR* = 0.422 (0.146–0.821), *p* = 0.011], additional prefire imaging [*OR* = 0.312 (0.143–0.684), *p* = 0.004] significantly increased diagnostic yield.

**Table 2:** Predictors for diagnostic failure of CT-guided TTNB in lung tumor ≤3 cm.

**Variables**	**Success (*n* = 851)**	**Failure (*n* = 108)**	***p***	Multivariable analysis OR (95%CI)^*^	***p***
Sex			0.975	–	
Female	432 (88.7%)	55 (11.3%)			
Male	419 (88.8%)	53 (11.2%)			
Age (years)^*^	62.65 ± 13.70	61.34 ± 14.09	0.350	–	
GGO part			0.667	–	
≦50%	786 (88.6%)	101 (11.4%)			
>50%	65 (90.3%)	7 (9.7%)			
Emphysema			0.460	–	
No	815 (88.9%)	102 (11.1%)			
Yes	36 (85.7%)	6 (14.3%)			
Obstructive pneumonitis			0.608	–	
No	843 (88.6%)	108 (11.4%)			
Yes	8 (100.0%)	0 (0%)			
Cavitary change			0.759	–	
No	827 (88.6%)	106 (11.4%)			
Yes	24 (92.3%)	2 (7.7%)			
Location			0.345	–	
LLL	139 (88.5%)	18 (11.5%)			
LUL	246 (90.1%)	27 (9.9%)			
RLL	179 (84.8%)	32 (15.2%)			
RML	62 (89.9%)	7 (10.1%)			
RUL	225 (90.4%)	24 (9.6%)			
Tumor depth (cm)	0.87 ± 0.95	0.88 ± 0.95	0.900	–	
Final pathology			<0.001		
Primary lung cancer					
Adenocarcinoma	461 (90.4%)	49 (9.6%)			
Squamous cell carcinoma	26 (83.9%)	5 (16.1%)			
Small cell carcinoma	10 (83.3%)	2 (16.7%)			
Others	6 (66.7%)	3 (33.3%)			
Metastasis	120 (81.1%)	28 (18.9%)			
Benign lung lesion	228 (94.2%)	14 (5.8%)			
**Coaxial**			0.773		**0.004**
No	484 (88.5%)	63 (11.5%)		1 (Reference)	
**Yes**	367 (89.1%)	45 (10.9%)		**0.340 (0.163**–**0.710)**	
Biopsy needle			0.768	–	
18-gauge	643 (88.6%)	83 (11.4%)			
20-gauge	208 (89.3%)	25 (10.7%)			
**Slice thickness**			0.027		**0.011**
**≦2.5 mm**	110 (94.8%)	6 (5.2%)		**0.422 (0.146**–**0.821)**	
>2.5 mm	741 (87.9%)	102 (12.1%)		1 (Reference)	
**Additional prefire imaging**			0.077		**0.004**
No	508 (87.3%)	74 (12.7%)		1 (Reference)	
**Yes**	343 (91.0%)	34 (9.0%)		**0.312 (0.143**–**0.684)**	
Tissue core number			0.195		0.833
≦2	734 (88.2%)	98 (11.8%)		1 (Reference)	
≧3	117 (92.1%)	10 (7.9%)		1.099 (0.456–2.650)	
Parenchymal tract distance			0.727	–	
≦2 cm	529 (88.5%)	69 (11.5%)			
>2 cm	322 (89.2%)	39 (10.8%)			
Transfissure			0.635	–	
No	841 (88.8%)	106 (11.2%)			
Yes	10 (83.3%)	2 (16.7%)			

*Unless otherwise indicated, data in parentheses are percentages.*.

* *Data are mean ± standard deviation*.

*The bold values are statistically significant in multivariate analysis, p < 0.05*.

In patients with lung tumor >3 cm, predictors for diagnostic success of CT-guided TTNB were shown in Table 3. In univariate analysis, coaxial needle usage (95.0 vs. 91.5%, *p* = 0.005), additional prefire imaging (95.1 vs. 91.5%, *p* = 0.005) significantly increased diagnostic yield; while GGO part more than 50% (72.7 vs. 93.4%, *p* = 0.003) and obstructive pneumonitis (89.1 vs. 94.4%, *p* < 0.001) significantly decreased diagnostic yield. In multivariate analysis, GGO part more than 50% [*OR* = 7.534 (2.806–20.225), *p* < 0.001] and obstructive pneumonitis [*OR* =2.310 (1.533–3.481), *p* < 0.001] significantly decreased diagnostic yield.

**Table 3:** Predictors for diagnostic failure of CT-guided TTNB in lung tumor >3 cm.

**Variables**	**Success (*n* = 1,487)**	**Failure (*n* = 110)**	***p***	Multivariable analysis OR (95%CI)^*^	***p***
Sex			0.055		
Female	647 (94.6%)	37 (5.4%)			
Male	840 (92.0%)	73 (8.0%)			
Age (years)^*^	64.80 ± 12.90	63.18 ± 12.88	0.205		
**GGO part**			0.003		**<0.001**
≦50%	1,471 (93.4%)	104 (6.6%)		1 (Reference)	
>50%	16 (72.7%)	6 (27.3%)		**7.534 (2.806–20.225)**	
Emphysema			0.112		
No	1,406 (93.4%)	100 (6.6%)			
Yes	81 (89.0%)	10 (11.0%)			
**Obstructive pneumonitis**			<0.001		**<0.001**
No	1,137 (94.4%)	67 (5.6%)		1 (Reference)	
Yes	350 (89.1%)	43 (10.9%)		**2.310 (1.533–3.481)**	
Cavitary change			0.824		
No	1,413 (93.1%)	104 (6.9%)			
Yes	74 (92.5%)	6 (7.5%)			
Location			0.283		
LLL	243 (92.0%)	21 (8.0%)			
LUL	422 (94.4%)	25 (5.6%)			
RLL	288 (92.6%)	23 (7.4%)			
RML	86 (88.7%)	11 (11.3%)			
RUL	448 (93.2%)	30 (6.3%)			
Tumor depth (cm)	0.53 ± 0.80	0.48 ± 0.85	0.604		
Final pathology			0.016		
Primary lung cancer					
Adenocarcinoma	950 (93.7%)	64 (6.3%)			
Squamous cell carcinoma	106 (87.6%)	15 (12.4%)			
Small cell carcinoma	54 (93.1%)	4 (6.9%)			
Others	12 (80.0%)	3 (20.0%)			
Metastasis	116 (91.3%)	11 (8.7%)			
Benign lung lesion	249 (95.8%)	11 (4.2%)			
Coaxial			0.005		0.833
No	796 (91.5%)	74 (8.5%)		1 (Reference)	
Yes	691 (95.0%)	36 (5.0%)		1.135 (0.351–3.662)	
Biopsy needle			0.638	–	
18-gauge	1,175 (93.0%)	89 (7.0%)			
20-gauge	312 (93.7%)	21 (6.3%)			
Slice thickness			0.129	–	
≧2.5 mm	180 (95.7%)	8 (4.3%)			
>2.5 mm	1,307 (92.8%)	102 (7.2%)			
Additional prefire imaging			0.005		0.384
No	821 (91.5%)	76 (8.5%)		1 (Reference)	
Yes	666 (95.1%)	34 (4.9%)		0.589 (0.179–1.939)	
Tissue core number			0.070	–	
≦2	1,177 (92.5%)	95 (7.5%)			
≧3	310 (95.4%)	15 (4.6%)			
Parenchymal tract distance			0.748	–	
≦2 cm	1,019 (93.0%)	77 (7.0%)			
>2 cm	468 (93.4%)	33 (6.6%)			
Transfissure			0.226	–	
No	1,466 (93.2%)	107 (6.8%)			
Yes	21 (87.5%)	3 (12.5%)			

*Unless otherwise indicated, data in parentheses are percentages*.

* *Data are mean ± standard deviation*.

*The bold values are statistically significant in multivariate analysis, p < 0.05*.

### Predictors for Tissue Adequacy

In cohort two, there were a total of 744 cases with a pathologic diagnosis of malignancy, and 31 cases (4.2%) were unsatisfactory for mutational analysis. A total of 333 biopsy specimens were sent for EGFR, 431 biopsy specimens were sent for ALK [ALK fluorescence *in situ* hybridization (FISH) was further done in 3 patients], 208 biopsy specimens were sent for ROS-1 (ROS1 FISH was further done in 7 patients) and 177 biopsy specimens were sent for PD-L1. Predictors for tissue adequacy were shown in Table 4. In univariate analysis, submission in two cassettes (98.9 vs. 94.9%, *p* = 0.027) was a predictor for tissue adequacy, while male (5.7 vs. 2.5%, *p* = 0.032), younger age (56.61 ± 11.64 vs. 65.82 ± 11.98, *p* < 0.001), and screening for clinical trial (18.5 vs. 0.7%, *p* < 0.001) significantly increased the risk of tissue inadequacy. Multivariate analysis was not performed due to multicollinearity of clinical trial, age and sex.

**Table 4:** Predictors for tissue adequacy of CT-guided TTNB.

**Variables**	**Adequate (*n* = 713)**	**Inadequate (*n* = 31)**	***p***
**Sex**			0.032
Female	347 (97.5%)	9 (2.5%)	
Male	366 (94.3%)	22 (5.7%)	
Age^*^	65.82 ± 11.98	56.61 ± 11.64	<0.001
GGO part			0.403
≦50%	673 (95.6%)	31 (4.4%)	
>50%	40 (100.0%)	0 (0%)	
Emphysema			1.000
No	694 (95.7%)	31 (4.3%)	
Yes	19 (100.0%)	0 (0%)	
Obstructive pneumonitis			0.533
No	565 (95.6%)	26 (4.4%)	
Yes	148 (96.7%)	5 (3.3%)	
Cavitary			1.000
No	686 (95.8%)	30 (4.2%)	
Yes	27 (96.4%)	1 (3.6%)	
Lobe			0.983
LLL	118 (95.1%)	6 (4.9%)	
LUL	205 (96.7%)	7 (3.3%)	
RLL	135 (95.7%)	6 (4.3%)	
RML	38 (95.0%)	2 (5.0%)	
RUL	217 (95.6%)	10 (4.4%)	
Tumor depth (cm)^*^	0.87 ± 0.95	0.86 ± 0.94	0.874
Tumor size			0.159
≦3 cm	235 (94.4%)	14 (5.6%)	
>3 cm	478 (96.6%)	17 (3.4%)	
**Clinical trial**			<0.001
No	594 (99.3%)	4 (0.7%)	
Yes	119 (81.5%)	27 (18.5%)	
Coaxial			0.687
No	296 (95.5%)	14 (4.5%)	
Yes	417 (96.1%)	17 (3.9%)	
Biopsy needle			0.630
18-gauge	422 (96.1%)	17 (3.9%)	
20-gauge	291 (95.4%)	14 (4.6%)	
Slice thickness			0.078
≦2.5 mm	221 (97.8%)	5 (2.2%)	
>2.5 mm	492 (95.0%)	26 (5.0%)	
Additional prefire imaging			0.297
No	323 (95.0%)	17 (5.0%)	
Yes	390 (96.5%)	14 (3.5%)	
Tissue core number			0.800
≦2	453 (96.0%)	19 (4.0%)	
≧3	260 (95.6%)	12 (4.4%)	
Tract distance (cm)^*^	5.49 ± 2.09	5.02 ± 1.60	0.223
**Submitted in two blocks**			0.027
No	541 (94.9%)	29 (5.1%)	
Yes	172 (98.9%)	2 (1.1%)	

*Unless otherwise indicated, data in parentheses are percentages*.

* *Data are mean ± standard deviation*.

### Complications

Pneumothorax and pulmonary hemorrhage are major complications of CT-guided TTNB. Logistic regression was used to identify risk factors for these procedure-related complications (Table 5). For pneumothorax, univariate analysis revealed that presence of emphysema along biopsy trajectory (62.4 vs. 35.5%, *p* < 0.001), tumor depth (0.94 ± 0.95 vs. 0.49 ± 0.78, *p* < 0.001), usage of 5 mm CT scan slice thickness (37.7 vs. 30.9%, *p* = 0.022), core tissue number ≤2 (38.1 vs. 31.2%, *p* = 0.006), parenchymal tract distance more than 2 cm (52.0 vs. 29.2%, *p* < 0.001), transfissural method (72.2 vs. 36.3%, *p* < 0.001) significantly increased the risk of pneumothorax. On the other hand, obstructive pneumonitis (17.5 vs. 40.5%, *p* < 0.001) significantly decreased the risk of pneumothorax. In multivariate analysis, male (*OR* = 1.26, *p* = 0.01, 95% CI = 1.06–1.50), presence of emphysema along needle trajectory (*OR* = 2.71, *p* < 0.001, 95% CI = 1.85–3.97), and tumor depth (*OR* = 1.40, *p* < 0.001, 95% CI = 1.24–1.58) significantly increased the risk of pneumothorax. In contrast, obstructive pneumonitis (*OR* = 0.53, *p* < 0.001, 95% CI = 0.39–0.71) and tumor size larger than 3 cm (*OR* = 0.6, *p* < 0.001, 95% CI = 0.5–0.73) significantly decreased the risk of pneumothorax. There were 3.2% (83/2,556) patients undergoing pleural drainage due to large amount of pneumothorax.

**Table 5:** Predictors for procedure-related complications.

**Variables**	**No pneumothorax (*n* = 1,614)**	**Pneumothorax (*n* = 942)**	***p***	**Multivariable analysis OR (95%CI)**	***p***	**No hemorrhage (*n* = 1,818)**	**Hemorrhage (*n* = 738)**	***p***	**Multivariable analysis OR (95%CI)**	***p***
**Sex**			0.012		0.010			0.045		0.376
Female	770 (65.8%)	401 (34.2%)		1 (Ref)		810 (69.2%)	361 (30.8%)		1 (Ref)	
Male	844 (60.9%)	541 (39.1%)		1.26 (1.06–1.50)		1,008 (72.8%)	377 (27.2%)		0.92 (0.76–1.11)	
Age^*^	63.79 ± 13.49	64.00 ± 12.86	0.703			63.97 ± 13.32	63.63 ± 13.10	0.557		
**GGO part**			0.465					<0.001		<0.001
≦50%	1,558 (63.3%)	904 (36.7%)				1,779 (72.3%)	683 (27.7%)		1 (Ref)	
>50%	56 (59.6%)	38 (40.4%)				39 (41.5%)	55 (58.5%)		2.83 (1.79–4.46)	
**Emphysema**			<0.001		<0.001			0.146		
No	1,564 (64.5%)	859 (35.5%)		1 (Ref)		1,716 (70.8%)	707 (29.2%)			
Yes	50 (37.6%)	83 (62.4%)		2.71 (1.85–3.97)		102 (76.7%)	31 (23.3%)			
**Obstructive pneumonitis**			<0.001		<0.001			<0.001		0.001
No	1,283 (59.5%)	872 (40.5%)		1 (Ref)		1,462 (67.8%)	693 (32.2%)		1 (Ref)	
Yes	331 (82.5%)	70 (17.5%)		0.53 (0.39–0.71)		356 (88.8%)	45 (11.2%)		0.54 (0.38–0.77)	
**Cavitary**			0.298					0.035		0.538
No	1,542 (62.9%)	908 (37.1%)				1,733 (70.7%)	717 (29.3%)		1 (Ref)	
Yes	72 (67.9%)	34 (32.1%)				85 (80.2%)	21 (19.8%)		0.85 (0.51–1.43)	
**Lobe**			0.061					0.005		0.036
LLL	254 (60.3%)	167 (39.7%)				320 (76.0%)	101 (24.0%)		1 (Ref)	
LUL	442 (61.4%)	278 (38.6%)				497 (69.0%)	223 (31.0%)		1.28 (0.96–1.72)	0.098
RLL	327 (62.6%)	195 (37.4%)				388 (74.3%)	134 (25.7%)		0.98 (0.72–1.34)	0.906
RML	95 (57.2%)	71 (42.8%)				123 (74.1%)	43 (25.9%)		1.05 (0.67–1.63)	0.841
RUL	496 (68.2%)	231 (31.8%)				490 (67.4%)	237 (32.6%)		1.41 (1.05–1.89)	0.021
**Tumor depth** (cm)	0.49 ± 0.78	0.94 ± 0.95	<0.001	1.40 (1.24–1.58)	<0.001	0.54 ± 0.80	0.94 ± 0.98	<0.001	1.21 (1.07–1.37)	0.002
**Tumor size**			<0.001		<0.001			<0.001		<0.001
≦3 cm	509 (53.1%)	450 (46.9%)		1 (Ref)		536 (55.9%)	423 (44.1%)		1 (Ref)	
>3 cm	1,105 (69.2%)	492 (30.8%)		0.60 (0.50–0.73)		1,282 (80.3%)	315 (19.7%)		0.39 (0.32–0.47)	
**Coaxial**			0.193					<0.001		0.419
No	879 (62.0%)	538 (38.0%)				951 (67.1%)	466 (32.9%)		1 (Ref)	
Yes	735 (64.5%)	404 (35.5%)				867 (76.1%)	272 (23.9%)		0.81 (0.49–1.35)	
**Biopsy needle**			0.737					0.965		
18-gauge	1,260 (63.3%)	730 (36.7%)				1,415 (71.1%)	575 (28.9%)			
20-gauge	354 (62.5%)	212 (37.5%)				403 (71.2%)	163 (28.8%)			
**Slice thickness**			0.022		0.151			<0.001		<0.001
≧2.5 mm	210 (69.1%)	94 (30.9%)		1 (Ref)		256 (84.2%)	48 (15.8%)		1 (Ref)	
>2.5 mm	1,404 (62.3%)	848 (37.7%)		1.29 (0.83–1.52)		1,562 (69.4%)	690 (30.6%)		2.16 (1.41–3.32)	
**Prefire imaging**			0.057					<0.001		0.741
No	911 (61.6%)	568 (38.4%)				996 (67.3%)	483 (32.7%)		1 (Ref)	
Yes	703 (65.3%)	374 (34.7%)				822 (76.3%)	255 (23.7%)		1.09 (0.65–1.82)	
**Tissue core number**			0.006		0.449			<0.001		0.714
≦2	1,303 (61.9%)	801 (38.1%)		1 (Ref)		1,444 (68.6%)	660 (31.4%)		1 (Ref)	
≧3	311 (68.8%)	141 (31.2%)		1.12 (0.83–1.52)		374 (82.7%)	78 (17.3%)		0.93 (0.65–1.35)	
**Parenchymal tract distance**			<0.001		<0.001			<0.001		<0.001
≦2 cm	1,200 (70.8%)	494 (29.2%)		1 (Ref)		1,311 (77.4%)	383 (22.6%)		1 (Ref)	
>2 cm	414 (48.0%)	448 (52.0%)		1.67 (1.35–2.06)		507 (58.8%)	355 (41.2%)		1.74 (1.38–2.19)	
**Transfissure**			<0.001		0.001			0.823		
No	1,604 (63.7%)	916 (36.3%)		1 (Ref)		1,793 (71.2%)	727 (28.8%)			
Yes	10 (27.8%)	26 (72.2%)		3.48 (1.62–7.47)		25 (69.4%)	11 (30.6%)			

For pulmonary hemorrhage, univariate analysis revealed that tumor with more than half GGO part (58.5 vs. 27.7%, *p* < 0.001), tumor location (*p* = 0.005, LUL or RUL showed higher rate of pulmonary hemorrhage), tumor depth (0.94 ± 0.98 vs. 0.54 ± 0.80, *p* < 0.001), usage of 5 mm CT scan slice thickness (30.6 vs. 15.8%, *p* < 0.001) significantly increased the risk of pulmonary hemorrhage in female. On the other hand, obstructive pneumonitis (11.2 vs. 32.2%, *p* < 0.001), presence of cavitary change (19.8 vs. 29.3%, *p* = 0.035), tumor size more than 3 cm (19.7 vs. 44.1%, *p* < 0.001), usage of coaxial needle (23.9 vs. 32.9%, *p* < 0.001), usage of additional prefire imaging (23.7 vs. 32.7%, *p* < 0.001), and obtaining more than two tissue core number (17.3 vs. 31.4%, *p* < 0.001) significantly decreased the risk of pulmonary hemorrhage. In multivariate analysis, tumor with GGO part more than 50% (*OR* = 2.83, *p* < 0.001, 95% CI = 1.79–4.46), tumor located in RUL (*OR* = 1.41, *p* = 0.021, 95% CI = 1.05–1.89), and tumor depth (*OR* = 1.21, *p* = 0.002, 95% CI = 1.07–1.37) significantly increased the risk of pulmonary hemorrhage. In contrast, obstructive pneumonitis (*OR* = 0.54, *p* = 0.001, 95% CI = 0.38–0.77) and tumor size larger than 3 cm (*OR* = 0.39, *p* < 0.001, 95% CI = 0.32–0.47) significantly decreased the risk of pulmonary hemorrhage.

The risk of air emboli was 0.3% (8/2,556). Three patients were symptomatic; one had acute stroke, one had acute myocardial infarction, and the other one had both acute stroke and myocardial infarction. Among these symptomatic patients, one had sequela of lock-in syndrome, and the other two had uneventful recovery. There was no significant predictor for symptomatic air emboli.

## Discussion

Accurate pathologic diagnosis is mandatory to determine appropriate treatment in patients with lung tumor. Additionally, there is increasing demand for specimens that fit the requirements for molecular characterization of lung tumor in order to accurately select patients who would benefit from target therapy and immunotherapy (11). Determining and predicting biopsy specimen accuracy and adequacy for the rapidly evolving molecular profiling techniques and assays are crucial (12, 13). Our findings suggested that tumor size more than 3 cm was a predictor for diagnostic success. This finding was consistent with prior studies showing that smaller lesions had higher diagnostic failure rate (1, 14, 15). CT scan slice thickness of 2.5 mm and additional prefire imaging significantly increased diagnostic yield. In addition, coaxial needle significantly increased the diagnostic yield.

As CT-guided TTNB is getting increasing popularity with the improvement of operator techniques and medical technology, the refinement of instruments furthers the precision in the diagnosis of small lung lesion. In a prior study, Tongbai et al. reported introducer needle outside the lesion as a predictor for a non-diagnostic biopsy (14). It is of crucial importance that the CT section thickness be appropriate to the lesion size. Partial volume averaging may cover up both the needle tip and the lesion in the same section or disguise as the tumor border, suggesting a seemingly correct needle path. In our study, coaxial needle significantly increased the diagnostic yield. In accordance with our result, Zhang et al. showed that coaxial technique promoted diagnostic accuracy of CT-guided TTNB for small (<1.5 cm) and deep (≥4 cm) lung lesions [95.5 vs. 72.7%, *p* = 0.023; (2)]. In the single-needle technique, if multiple tissue samples were requested, then multiple passes are needed, which prolonged the procedure and increased radiation exposure. The single-needle technique causes patient discomfort and increases the risk of complications such as hemorrhage or pneumothorax with each needle pass. In contrast, biopsy cutting needles can be advanced in a coaxial fashion through the guide needle in coaxial-needle technique. The guide needle allows rapid sampling of the tumor using multiple biopsy needles without additional imaging, additional discomfort, or risk to the patient. It also allows acquisition of larger tissue volume, with repeated biopsies at areas of different depths using the outer coaxial needle.

Prior studies showed that final benign diagnosis was a risk factor for biopsy failure (14, 16). According to Tongbai et al., among patients with malignancy as the final diagnosis, lymphoproliferative disease had a higher rate of diagnostic failure than primary lung cancer [29% (5/17) vs. 5% (34/631)] (14). In our study, primary lung squamous cell carcinoma, certain rare primary lung cancer, and pulmonary metastasis rather than adenocarcinoma contributed to the population with most difficult diagnosis. The lung squamous cell carcinoma mainly involves the bronchi and sometimes it is difficult to distinguish the tumor itself from the downstream ventilation disorder, which contribute to possible cause of diagnostic failure. For these rapid growing tumors, extensive tumor necrosis may be seen under microscope even when avid enhancing part of the tumor is selected as the biopsy target. For this situation, rapid on-site evaluation (ROSE) in CT room or surgical biopsy would improve diagnostic yield.

In our study, using a coaxial needle and additional prefire imaging increased overall diagnostic yield. In the subgroup analysis, for lesions smaller than or equal to 3 cm, using a coaxial needle and CT slice thickness <2.5 mm with prefire imaging significantly increased diagnostic success rate. Confirming specimen notch within the lesion was important to increase the accuracy of biopsy for smaller lesion. On the contrary, for lesions larger than 3 cm, characteristics of lesions (GGO part or obstructive pneumonitis) played a vital role in diagnostic yield. Predominant GGO tumor implied less invasiveness, and pathologist might only be able to make the diagnosis of AAH based on limited core tissue specimen. The presence of obstructive pneumonitis made it difficult to identify the exact location of the tumor and hence increased the diagnostic failure rate.

In the era of precision medicine, adequate biopsy specimens are paramount in providing direct source to investigate the molecular biomarkers, helping both cancer diagnosis and treatment. A prior study by Jamshidi et al. indicated that in order to optimize nucleic acid yields for genomic analysis in CT-guided TTNB, needles of lower gauge are more preferable than higher gauge (12). According to Beck et al., four samplings with 20-gauge coaxial needle is adequate for various molecular studies for lung malignancy (17). Our results showed that putting the core specimens in two separately numbered formalin containers rather than one formalin container was more efficient in tissue preservation. It was in consistent with Ferguson et al. reporting multidisciplinary approach to optimize tissue volume in CT-guided TTNB (18). Although needles of lower gauge and increased number of core tissue increase specimen amount, diagnostic tissue loss is a major problem during repetitive cutting at the microtome. Our study result showed that separating the specimen into two cassettes was more important than obtain more biopsy specimens. Saving tissue ribbons on unstained slides could help tissue preservation for future IHC stains, FISH, or molecular studies. Placing biopsy material in two tissue cassettes could decrease the rate of tissue inadequacy by leaving the second block available for molecular testing, and could be routinely adopted in the future. In accordance with our result, Beck et al. reported a success rate of 96% (96/100) in patients with lung malignancy undergoing five molecular studies (EGFR mutation, ALK translocation, KRAS mutation, RET and ROS1 rearrangements) by placing each core from 20-gauge needle in separate formalin containers. The average core biopsy samples for molecular analysis of adequate group and inadequate group were 4.1 (range, 1–9) vs. 3.3 (range, 1–5), respectively (17).

Pneumothorax is the most common complication in CT-guided TTNB, ranging from 8.4 to 42.3% in prior meta-analysis study (19). Our results demonstrated a pneumothorax rate of 36.9% and pneumothorax requiring drainage tube insertion in 3.2% of the total cohort. Our study showed that presence of emphysema along needle trajectory, tumor depth, parenchymal tract distance, and transfissure trajectory significantly increased the risk of pneumothorax. These findings were in accordance with prior studies. Lee et al. also reported perilesional emphysema is a predictor for pneumothorax [*OR* = 3.720, *p* < 0.001, 95% CI = 3.265–13.831; (20)]. Huang et al. reported that longer parenchymal tract distance increased the risk of pneumothorax [21.5 ± 18.3 vs. 15.6 ± 16.5, *p* = 0.002; (1)]. Elshafee et al. showed that emphysema (*OR* = 8.8, *p* < 0.001), lesion depth from the pleura (*OR* = 1.9, *p* < 0.001), and fissure puncture (*OR* = 9.4, *p* = 0.01) significantly increased the risk of pneumothorax in patients undergoing CT-guided TTNB using non-coaxial semi-automated 18 gauge biopsy system (21). Our result showed obstructive pneumonitis (*OR* = 0.53, *p* < 0.001, 95% CI = 0.39–0.71) and tumor size larger than 3 cm (*OR* = 0.60, *p* < 0.001, 95% CI = 0.50–0.73) significantly decreased the risk of pneumothorax, which had not been reported in prior study.

Prior meta-analysis showed post-procedure pulmonary hemorrhage rated from 2.9 to 50.8% (19). Our results demonstrated a pulmonary hemorrhage rate of 28.9%. Li et al. showed that tumor depth was an independent risk factor for grade 2 or higher hemorrhage in patients undergoing CT-guided TTNB for small (≤20 mm) lung nodules [*p* = 0.024; (22)]. This is in accordance with our study finding. Lee et al. reported perilesional emphysema was associated with higher rate of hemorrhage [*OR* = 3.877, *p* = 0.001, 95% CI = 1.796–8.367; (20)], but we did not have similar finding.

Recently published data by Lee et al. showed that pooled incidence of symptomatic air embolism after TTNB was 0.08% (95% CI, 0.048–0.128%), and one-third of cases had sequelae or died (23). The finding was in concordance with our result, where 0.12% (3/2,556) of our study cohort had symptomatic air emboli. Lee et al. indicated that the presence of an underlying disease (OR, 5.939; 95% CI, 1.029–34.279; *p* = 0.046), the use of a ≥19-gauge needle (OR, 10.046; 95% CI, 1.103–91.469; *p* = 0.041), and coronary or intracranial air embolism (OR, 19.871; 95% CI, 2.725–14.925; *p* = 0.003) were independent risk factors for symptomatic embolism. Unfavorable outcomes were independently associated with the use of aspiration biopsy rather than core biopsy (OR, 3.302; 95% CI, 1.149–9.492; *p* = 0.027) and location of the air embolism in the coronary arteries or intracranial spaces (*OR* = 5.173; 95% CI = 1.309–20.447; *p* = 0.019). However, in our study, we did not find any significant predictor for symptomatic air emboli.

The study results provide real world data for imaging-guided interventionalists involved in tissue acquisition for genomic-based personalized medicine. Because the diagnostic yield and quality of the samples determine the utility of the obtained tissue, procedural planning to ensure adequate amount of tissue biopsy specimens is important. Our results suggested the use a coaxial needle, thin CT slice thickness (2.5 mm), additional prefire imaging, and obtaining more than two tissue cores and submitting in two cassettes (Supplementary Figure 1).

Our study had limitations. First, it was a retrospective and single center study. Second, the biopsy method was based on the discretion of the operator, which could lead to selection bias. Third, we had a long study period. Therefore, the indication, technique of biopsy and experience of operators may have changed over the years. The operator subjectively decided the biopsy method, which will be an unneglectable bias. Since we did not perform subgroup analysis for treatment naïve and treatment failure groups, the post-treatment lung injury or tumor heterogeneity might influence diagnostic yield and complications. Future larger prospective studies are warranted to validate our study findings.

## Conclusion

In conclusion, using a coaxial needle, slice thickness of 2.5 mm, and additional prefire imaging improved diagnostic success. Obtaining more than two tissue cores and submitting in two cassettes improved tissue adequacy for molecular testing, and hence may optimize patient treatment.

## Data Availability Statement

The original contributions presented in the study are included in the article/Supplementary Material, further inquiries can be directed to the corresponding author/s.

## Ethics Statement

The studies involving human participants were reviewed and approved by National Cheng Kung University Hospital. Written informed consent for participation was not required for this study in accordance with the national legislation and the institutional requirements.

## Author Contributions

C-YL and C-CC were involved in data collection, study design, analysis, preparation, and review of manuscript. C-YC, L-TH, and T-JC were involved in data collection and review of manuscript. Y-SL and Y-TY were involved in review of manuscript. All authors contributed to the article and approved the submitted version.

## Conflict of Interest

The authors declare that the research was conducted in the absence of any commercial or financial relationships that could be construed as a potential conflict of interest.

## Supplementary Material

Supplementary Figure 1 Illustrations of important factors improve diagnostic success and tissue adequacy: using a coaxial needle, obtaining additional prefire imaging, with thin CT slice thickness (2.5 mm), obtaining more than two tissue cores and submitting in two cassettes.

## Abbreviations

CT: computed tomography
TTNB: transthoracic needle biopsy
OR: odds ratio
AIS: adenocarcinoma *in situ*
GGO: ground glass opacity
AAH: atypical adenomatous hyperplasia
IHC: immunohistochemistry
EGFR: epidermal growth factor receptor.
