# Supplementary figures and images for: Computed Tomography-Guided Transthoracic Needle Biopsy: Predictors for Diagnostic Failure and Tissue Adequacy for Molecular Testing
Source: Front Med (Lausanne). 2021 May 19;8:650381. doi: 10.3389/fmed.2021.650381 (PMC8169979; doi:10.3389/fmed.2021.650381)

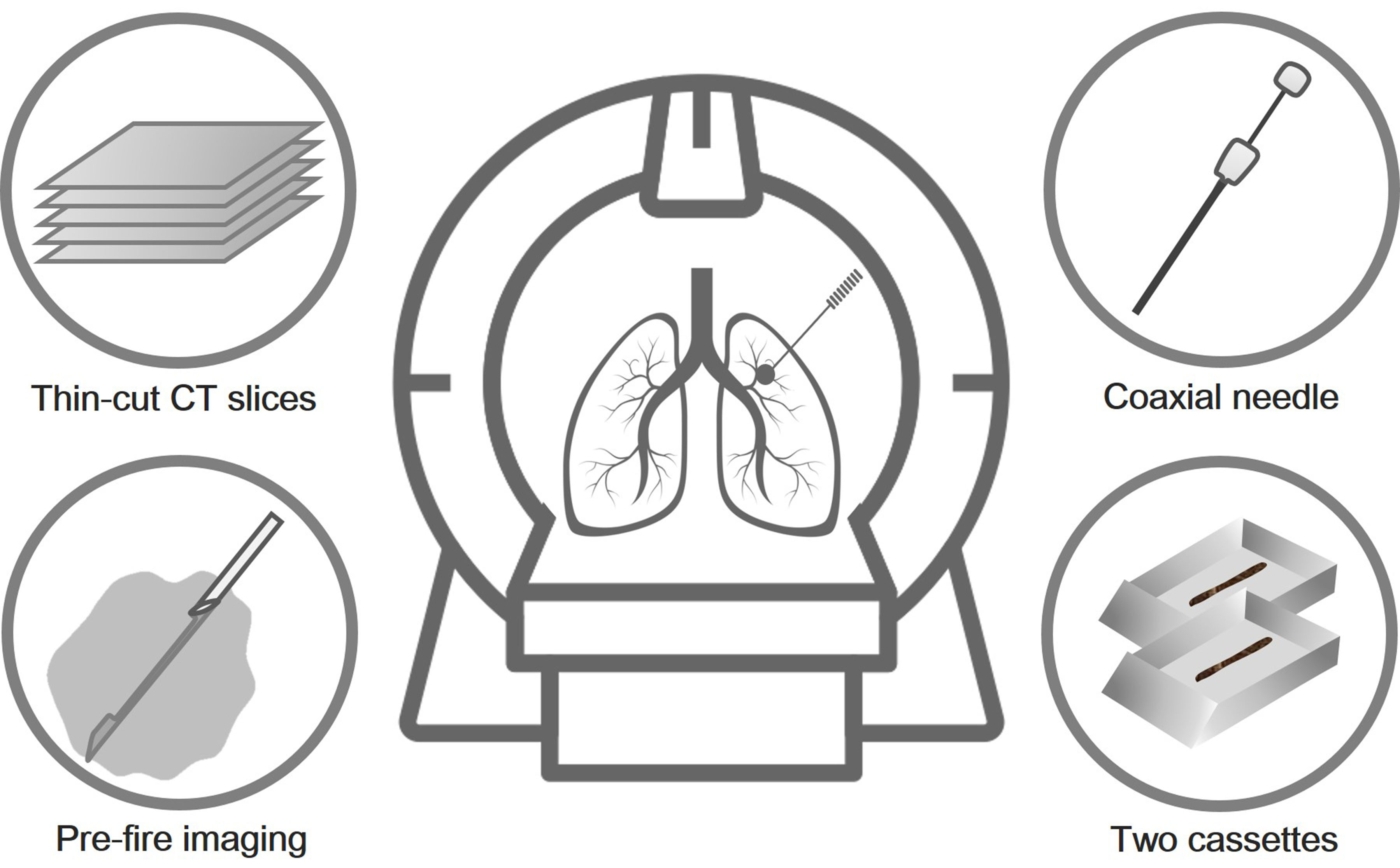

Supplement: Supplementary Figure 1 — Illustrations of important factors improve diagnostic success and tissue adequacy: using a coaxial needle, obtaining additional prefire imaging, with thin CT slice thickness (2.5 mm), obtaining more than two tissue cores and submitting in two cassettes. [file Image_1.JPEG]
